# *De Novo* Assembly and Characterization of the Transcriptome, and Development of SSR Markers in Wax Gourd (*Benicasa hispida*)

**DOI:** 10.1371/journal.pone.0071054

**Published:** 2013-08-08

**Authors:** Biao Jiang, Dasen Xie, Wenrui Liu, Qingwu Peng, Xiaoming He

**Affiliations:** 1 Vegetable Research Institute, Guangdong Academy of Agricultural Science, Guangzhou, China; 2 Guangdong Provincial Key Lab for New Technology Research on Vegetables, Guangzhou, China; Kyushu Institute of Technology, Japan

## Abstract

**Background:**

Wax gourd is a widely used vegetable of *Cucuribtaceae*, and also has important medicinal and health values. However, the genomic resources of wax gourd were scarcity, and only a few nucleotide sequences could be obtained in public databases.

**Methodology/Principal Findings:**

In this study, we examined transcriptome in wax gourd. More than 44 million of high quality reads were generated from five different tissues of wax gourd using Illumina paired-end sequencing technology. Approximately 4 Gbp data were generated, and *de novo* assembled into 65,059 unigenes, with an N50 of 1,132 bp. Based on sequence similarity search with known protein database, 36,070 (55.4%) showed significant similarity to known proteins in Nr database, and 24,969 (38.4%) had BLAST hits in Swiss-Prot database. Among the annotated unigenes, 14,994 of wax gourd unigenes were assigned to GO term annotation, and 23,977 were found to have COG classifications. In addition, a total of 18,713 unigenes were assigned to 281 KEGG pathways. Furthermore, 6,242 microsatellites (simple sequence repeats) were detected as potential molecular markers in wax gourd. Two hundred primer pairs for SSRs were designed for validation of the amplification and polymorphism. The result showed that 170 of the 200 primer pairs were successfully amplified and 49 (28.8%) of them exhibited polymorphisms.

**Conclusion/Significance:**

Our study enriches the genomic resources of wax gourd and provides powerful information for future studies. The availability of this ample amount of information about the transcriptome and SSRs in wax gourd could serve as valuable basis for studies on the physiology, biochemistry, molecular genetics and molecular breeding of this important vegetable crop.

## Introduction

The *Cucurbitaceae* is an important family in the plant kingdom, whose importance is just after *Gramineae*, *Leguminosae* and *Solanaceae*. Over the past several years, the genome sequencing of many crops in *Cucurbitaceae* has been completed, such as cucumber [1], melon [2], and watermelon [3]. However, the genomic information about wax gourd ( *Benincasa* *hispida* (Thunb.) Cogn (2n=2*x*=24).) is lacking. The wax gourd, also called white gourd, white pumpkin, tallow gourd, ash gourd, and so on, is named after the Italian count, Giuseppe Benincasa [4]. It is a monotypic genus which belongs to the family *Cucurbitaceae*, and is a widely used vegetable in India, China and other tropical countries [5]. Fruit is the edible organs of wax gourd, and often consumed as baked, fried, boiled, pickled or candied/preserved. Since the storage of wax gourd is very long, it plays an important role in ensuring the annual supply and regulating off-seasons of the vegetables. In addition, wax gourd is recommended for treatment of peptic ulcer, hemorrhages from internal organs, epilepsy and other neurological disorders [6,7]. It is also reported that the fresh juice is effective in preventing morphine withdrawal in mice [8]. In spite of its high economic importance, the research on wax gourd, particularly at the levels of molecular biology and genetics, is very weak. The studies on wax gourd, to data, are only confined to a few fields, such as genetic diversity [9,10], and drug development [6,7,11]. The genomic information of wax gourd is very limited. Up to currently, there are only 39 nucleotide sequences submitted in the NCBI nucleotide database. In addition, there is no simple sequence repeat (SSR) marker developed in wax gourd.

Transcriptome is the complete collection of transcripts in a cell at a specific developmental stage, which provides valuable and comprehensive information on gene expression, gene regulation, and amino acid content of proteins. The development of sequencing technologies, such as Illumina paired-end sequencing technology, has provided a novel method for the analyses of transcriptome [12]. Such technology has been successfully applied ubiquitously in both model plants and non-model species. In the *Cucurbitaceae* family, there are many reports of transcriptome sequencing, such as *Cucumis sativus* [13], *C. melo* [14], *Citrullus lanatus* [15], *Momordica* *cochinchinensis* [16] and so on. The analysis of transcriptome is of great importance for gene annotation and discovery, comparative genomics, and development of molecular markers [14,17-22]. For example, in *C. melo*, its transcriptome was sequenced for SNP discovery [14]. Using the Illumina paired-end sequencing technology, the transcriptomes of the immature seeds were analyzed and 3, 919 microsatellite markers were developed in peanut [19].

In order to obtain the comprehensive genomic information of wax gourd, we performed the present study, aiming to acquire the detailed transcriptome profile of wax gourd by utilizing Illumina paired-end sequencing technology and to develop SSR markers based on the transcriptome sequences for subsequent studies of wax gourd at levels of physiology, biochemistry, molecular biology and genetics.

## Results

### Sequencing and de novo assembly of Illumina paired-end reads

In order to obtain a broad survey of genes associated with the growth and development of wax gourd, total RNA samples were extracted from shoot tips, leaves, flowers, fruits and stems in the flowering stage. The samples were sequenced using Illumina paired-end sequencing technology. SOAPdenovo, which was developed specifically for next-generation short-read sequences, was used for *de novo* assembly. When stringent quality was check and data were cleaned, about 44, 925, 792 high quality reads were obtained with 96.1% of Q20 bases (base quality more than 20). Based on the high quality reads, a total of 66, 129 of contigs were assembled, with total nucleotides of 49, 918, 791 bp, and an N50 of 1, 131 bp (i.e. 50% of the assembled bases were incorporated into contigs of 1, 131 bp). The length of contigs ranged from 201 bp to 11, 829 bp, with an average of 755 bp. Using the Trinity assembling program, the de novo assembly yielded 65, 059 unigenes, with an N50 of 1, 132 bp. The average length of unigenes was 709 bp (Table 1).

**Table 1:** Summary for the transcriptome of wax gourd.

	Total number		Total Nucleotides base (bp)										Average length (bp)										N50	
Reads	44, 925, 792		4, 043, 321, 280										90										−	
Contigs	66, 129		49, 918, 791										755										1, 131	
Unigenes	65, 059		46, 146, 322										709										1, 132	

### Evaluation of *de novo* assembly

**Figure 1:**
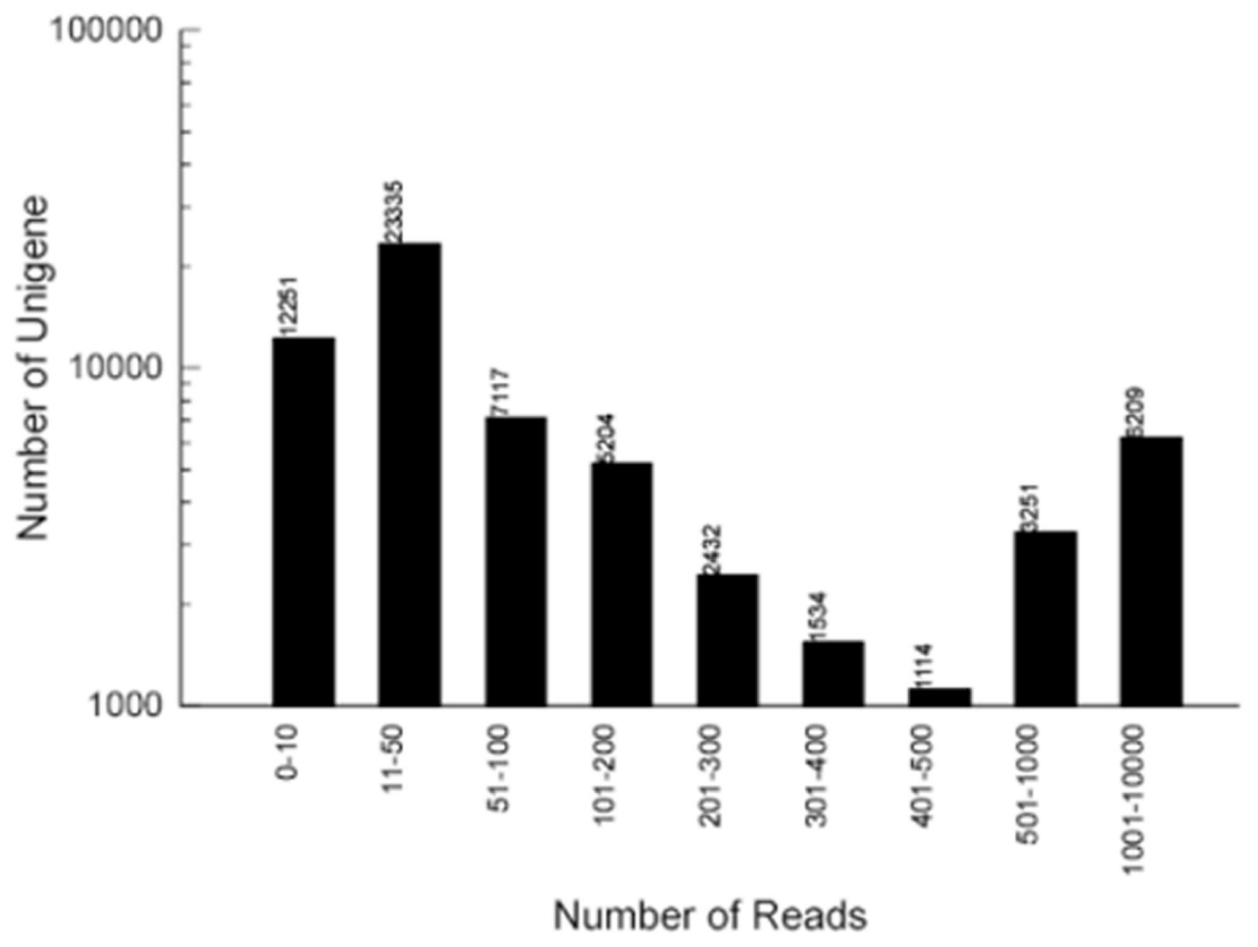
Assessment of assembly quality. Distribution of unique-mapped reads of the assembled unigenes.

**Figure 2:**
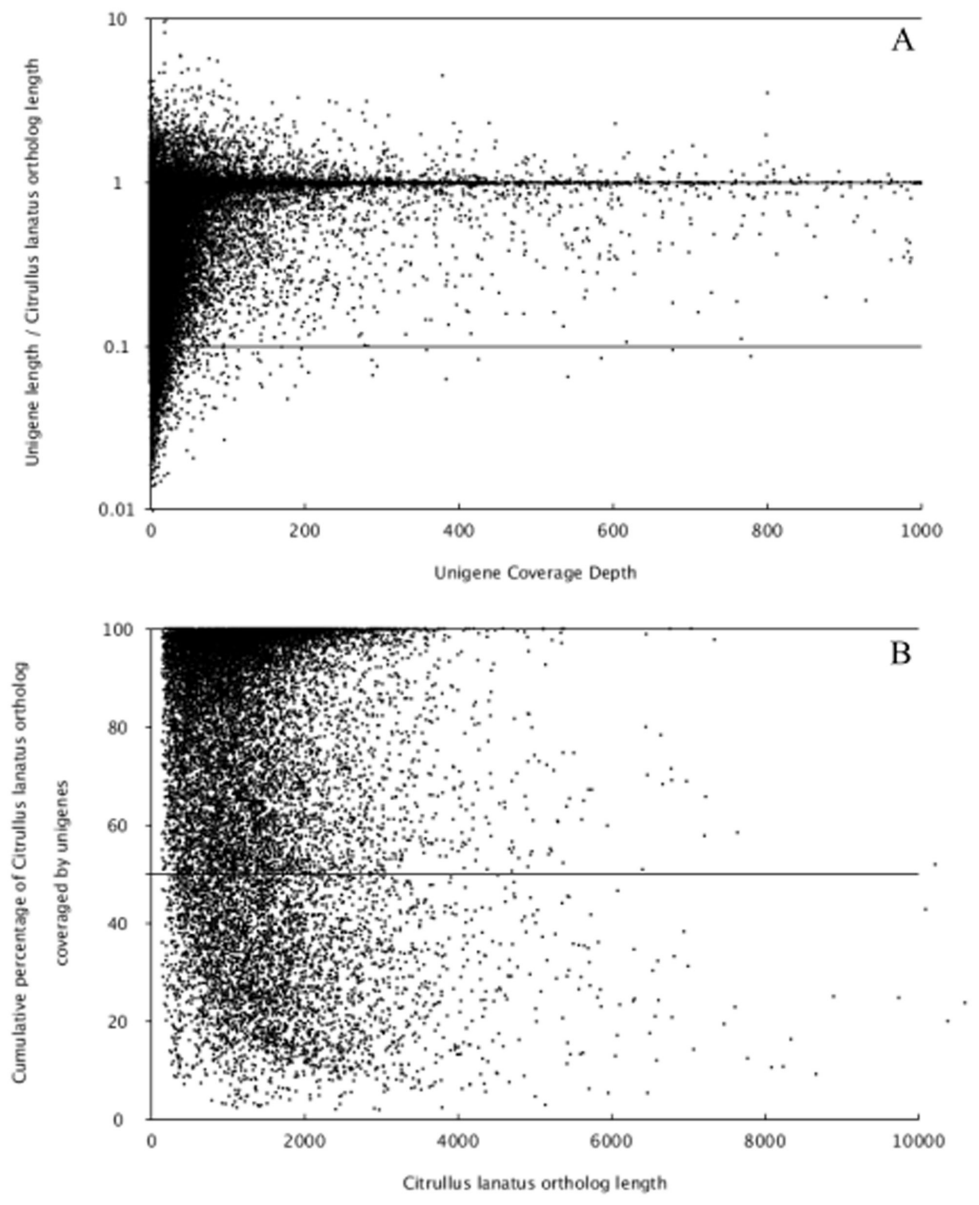
Comparison of wax gourd unigenes to orthologous *C. lanatus* coding sequences. (A) The ratio of wax gourd unigene length to *C. lanatus* ortholog length was plotted against wax gourd unigene coverage depth. Overall, there are 3, 227 unigenes with the ratio greater than 1, and 25, 763 unigenes with the ratio less than 1. (B) Total percent of *C. lanatus* ortholog coding sequence that was covered by all wax gourd unigenes. In total, 8, 936 orthologs could be covered by unigenes with a percentage of more than 80%, and the cover percentage of around 4, 796 orthologs ranged from 40% to 80%. Furthermore, 827 orthologs were covered with only 20% or lower.

To evaluate the quality and coverage of the assembled unigenes, SOAPaligner, which allowed up to 2 base mismatches, was employed to realign all the usable sequencing reads to the unigenes [23]. The sequencing depth ranged from 0.03 to 20, 359 folds, with an average of 44.25 folds. About 80.4%, 31.6% and 10.0% of the unigenes were realigned by more than 10 reads, more than 100 reads and more than 1000 reads, respectively (Figure 1). Furthermore, in order to assess the extent of transcript coverage provided by unigenes and to evaluate how coverage depth affected the unigene assembly, the ratio of the assembled unigene length to *Citrullus lanatus* ortholog length against coverage depth was calculated (Figure 2A). Although a large number of deeply covered unigenes failed to cover the complete coding regions of their *C. lanatus* orthologs, our unigenes can cover most of *C. lanatus* orthologs. To certain extent, increased coverage depth can result in higher coverage of the coding regions. In our present study, there are 3, 227 unigenes with the ratio greater than 1, and 25, 763 unigenes with the ratio less than 1. The percentage of *C. lanatus* ortholog coding sequence that was covered by all wax gourd unigenes was also performed. In total, 8, 936 orthologs could be covered by unigenes with a percentage of more than 80%, and the coverage percentage of around 4, 796 orthologs ranged from 40% to 80%. Furthermore, 827 orthologs were covered with only 20% or lower (Figure 2B).

### Functional annotation

**Figure 3:**
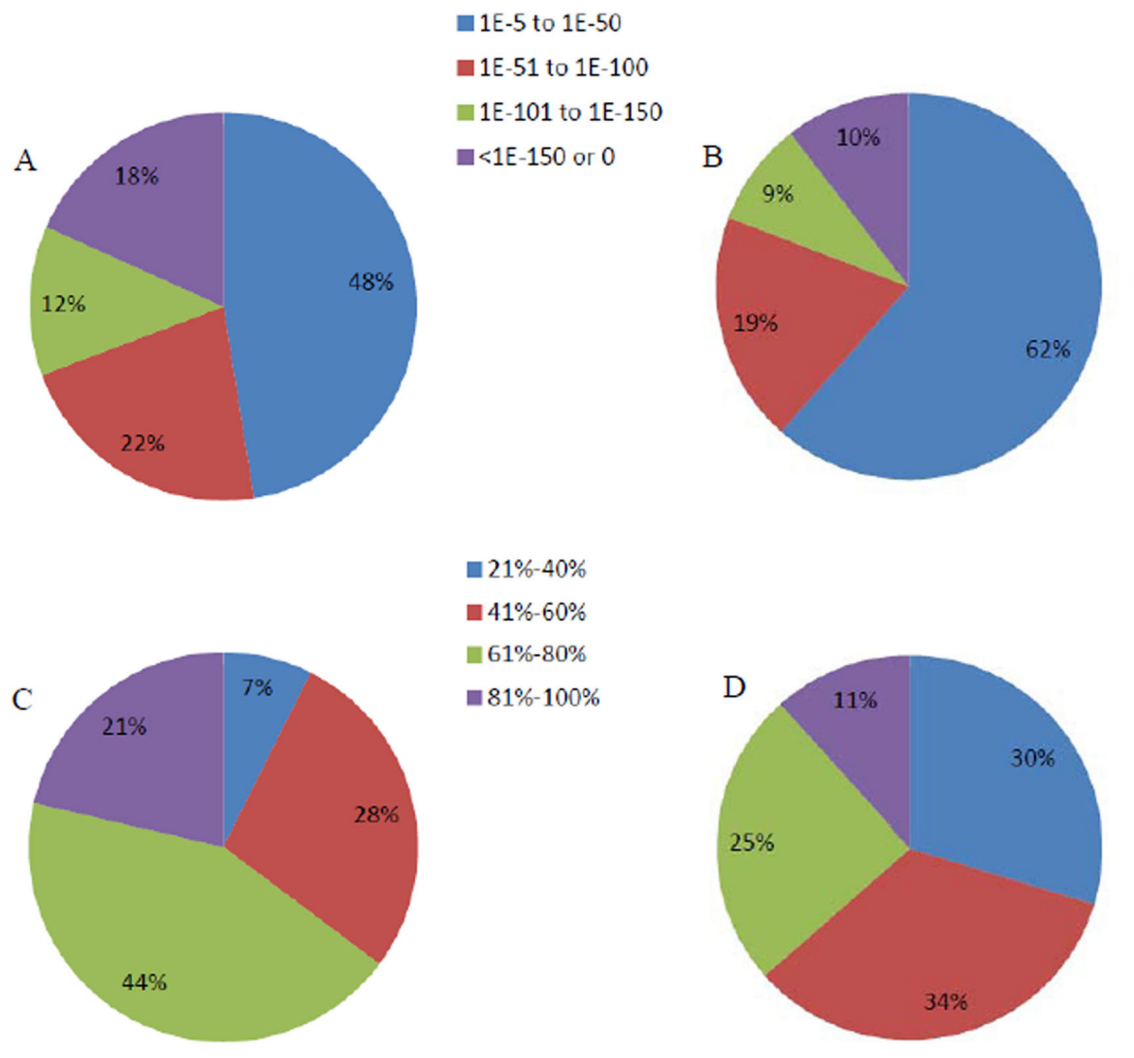
Characteristics of similarity search of unigenes against Nr and Swiss-Prot databases. (A) E-value distribution of BLAST hits for each unigene with an E-value threshold of 10^-5^ in Nr database. (B) E-value distribution of BLAST hits for each unigene with an E-value threshold of 10^-5^ in Swiss-Prot database. (C) Similarity distribution of the top BLAST hits for each unigene in Nr database. (D) Similarity distribution of the top BLAST hits for each unigene in Swiss-Prot database.

A sequence similarity search was conducted against the NCBI non-redundant protein (Nr) database, the Swiss-Prot protein database, using BLASTx algorithm with an E-value threshold of 10^-5^. The results showed that out of 65, 059 unigenes, 36, 070 (55.4%) showed significant similarity to known proteins in Nr database, and 24, 969 (38.4%) had BLAST hits in Swiss-Prot database. The E-value distribution of the top hits in the Nr database revealed that 52.0% of the mapped unigenes showed significant homology with the E-value less than 1E-50, and there were 21.0% of the unigenes with similarity great than 80% (Figure 3A and 3C). On the other hand, the E-value and similarity distributions of the top hits in Swiss-Prot database had a comparable pattern with 38.0% and 11.0% of the unigenes possessing significant homology and similarity, respectively (Figure 3B and 3D).

Based on Nr annotation, 14, 994 unigenes were assigned gene ontology (GO) terms with 68, 590 functional terms. GO-annotated unigenes had three ontologies, including biological process, cellular component, and molecular function. Among them, the unigenes for cellular components made up the majority (27, 253, 44.1%), followed by the unigenes for biological process (26, 507, 42.9%), and for molecular function (8, 067, 13.1%) (Table 2). The functionally assigned unigenes covered a comprehensive range of GO categories. Under the cellular component category, cell component (9, 568, 35.1%), cell part component (8, 515, 31.2%) and organelle component (6, 466, 23.7%) represented the majorities, whereas only a few unigenes were assigned to cell junction (3), extracellular region part (6), and extracellular region (11). Under the biological process category, metabolic process (8, 801, 25.7%) and cellular process (6, 752, 25.5%) were prominently represented. Furthermore, 2, 264 unigenes are involved in response to different stimulus. For the molecular function category, catalytic activity (6, 763, 45.6%) and binding (6, 716, 45.3%) represented the majorities.

**Table 2:** Gene Ontology classification of assembled unigenes.

Ontology	Class	Number of unigene
Biological process	Biological adhesion	2
	Biological regulation	1262
	Cellular component organization or biogenesis	1034
	Cellular process	6752
	Death	100
	Developmental process	1240
	Establishment of localization	1362
	Growth	99
	Immune system process	77
	Localization	1503
	Locomotion	4
	Metabolic process	6801
	Multi-organism process	239
	Multicellular organismal process	898
	Negative regulation of biological process	122
	Nitrogen utilization	2
	Pigmentation	2
	Positive regulation of biological process	39
	Regulation of biological process	968
	Reproduction	590
	Reproduction process	577
	Response to stimulus	2264
	Rhythmic process	11
	Signaling	554
	Viral reproduction	5
Cellular component	Cell	9568
	Cell junction	3
	Cell part	8515
	Extracellular region	11
	Extracellular region part	6
	Macromolecular complex	881
	Membrane-enclosed lumen	227
	Organelle	6466
	Organelle part	1576
Molecular function	Antioxidant activity	19
	Binding	6716
	Catalytic activity	6763
	Enzyme regulator activity	82
	Molecular transducer activity	346
	Protein binding transcription factor activity	19
	Receptor activity	45
	Transporter activity	840

The results were summarized in three main categories: biological process, cellular component and molecular function.

**Figure 4:**
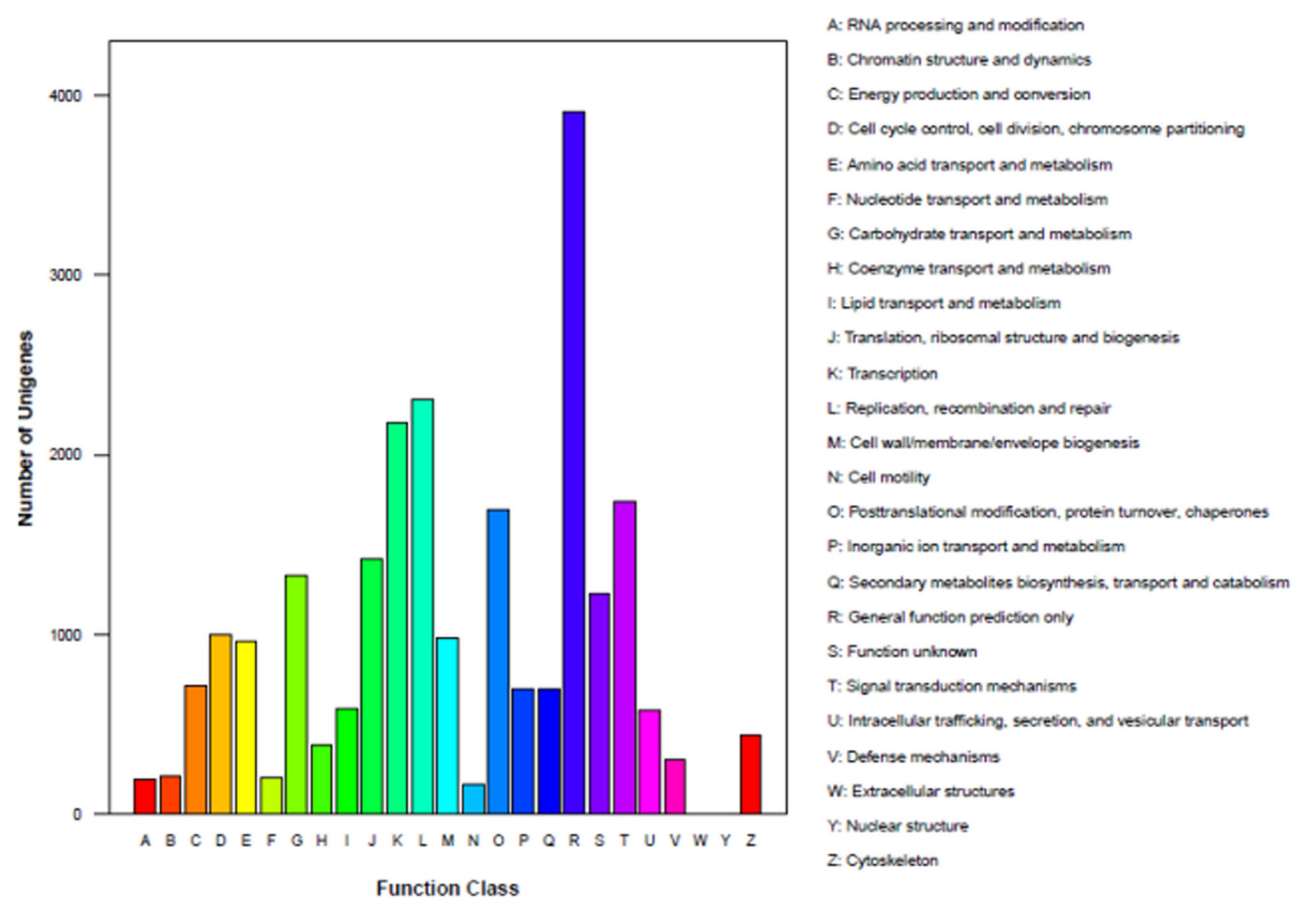
Clusters of orthologous groups (COG) classification of wax gourd transcriptome. All the unigenes were aligned to COG database to predict and classify possible functions. In total 23, 977 of the 36, 070 unigenes with Nr hits were grouped into 25 COG classifications.

Furthermore, all unigenes were subjected to a search against the Cluster of Orthologous Groups (COG) database for functional prediction and classification. Overall, 23, 977 of the 36, 070 unigenes showing Nr hits were assigned to COG classifications (Figure 4). Among the 25 COG categories, the cluster for general function prediction only represented the largest one (3, 908, 16.3%), followed by replication, recombination and repair (2, 313, 9.7%), transcription (2, 177, 9.1%), signal transduction mechanisms (1, 743, 7.3%), posttranslational modification, protein turnover, chaperones (1, 695, 7.1%), translation, ribosomal structure and biogenesis (1, 418, 5.9%), carbohydrate transport and metabolism (1, 328, 5.5%), function unknown (1, 231, 5.1%) and cell cycle control, cell division, chromosome partitioning (1, 003, 4.2%), whereas only a few unigenes were assigned to extracellular structures and nuclear structure (6 and 1 unigenes, respectively).

To identify the biological pathways in wax gourd, the annotated unigenes were mapped to the reference of typical pathways in the Kyoto Encyclopedia of Genes and Genomes (KEGG) [24]. A total of 18, 713 unigenes had significant matches in the KEGG database and were assigned to 281 KEGG pathways. Among them, the metabolic pathway containing 4, 274 unigenes is the largest one, followed by biosynthesis of secondary metabolites (1,953), plant hormone signal transduction (1,258), and plant/pathogen interaction (1,186). It is worth noting that 91 unigenes were assigned to the biosynthesis of unsaturated fatty acids. Furthermore, there were also many unigenes mapped to vitamin B6 metabolism, arachidonic acid metabolism, flavonoid biosynthesis, phenylalanine metabolism, and so on.

### Development of SSR markers in wax gourd

SSRs are useful as molecular markers for genetics and biology researches. In order to develop SSR markers in wax gourd, all the 65, 059 unigenes generated in our present study were used to mine potential microsatellites, which were defined as di- to hexa-nucleotide SSR with a minimum of four repeats for all motifs (except for di-nucleotide with a minimum of six repeats, and tri-nucleotide with a minimum of five repeats). Finally, 6, 242 microsatellites were detected in 5, 416 unigenes, out of which, 694 unigenes contained more than 1 SSRs. Among the microsatellites, tri-nucleotide motifs were the most abundant types (2, 846, 45.6%), followed by di-nucleotide (2, 447, 39.2%), tetra-nucleotide (530, 8.5%), penta-nucleotide (221, 3.5%) and hexa-nucleotide (198, 3.2%) motifs (Table 3). Furthermore, the length of SSRs was also analyzed, which was mainly distributed from 12 to 20 bp, accounting for 86.7% of the total SSRs (Table 4).

**Table 3:** Summary of SSR mining results.

Search item	Numbers
Total number of sequences examined	65, 059
Total size of examined sequences (bp)	46, 146, 322
Total number of identified SSRs	6, 242
Number of unigenes containing SSRs	5, 416
Number of unigenes containing more than 1 SSR	694
Number of SSRs present in compound formation	291
Di-nucleotide	2, 447
Tri-nucleotide	2, 846
Tetra-nucleotide	530
Penta-nucleotide	221
Hexa-nucleotide	198

**Table 4:** Summary of the number of repeat units.

Number of repeat unit	Di-	Tri-	Tetra-	Penta-	Hexa-
4	0	0	383	200	187
5	0	1, 541	127	20	9
6	924	840	12	0	2
7	582	427	2	0	0
8	363	36	5	1	0
9	256	2	0	0	0
10	193	0	0	0	0
11	116	0	0	0	0
12	12	0	1	0	0
≥13	1	0	0	0	0

**Figure 5:**
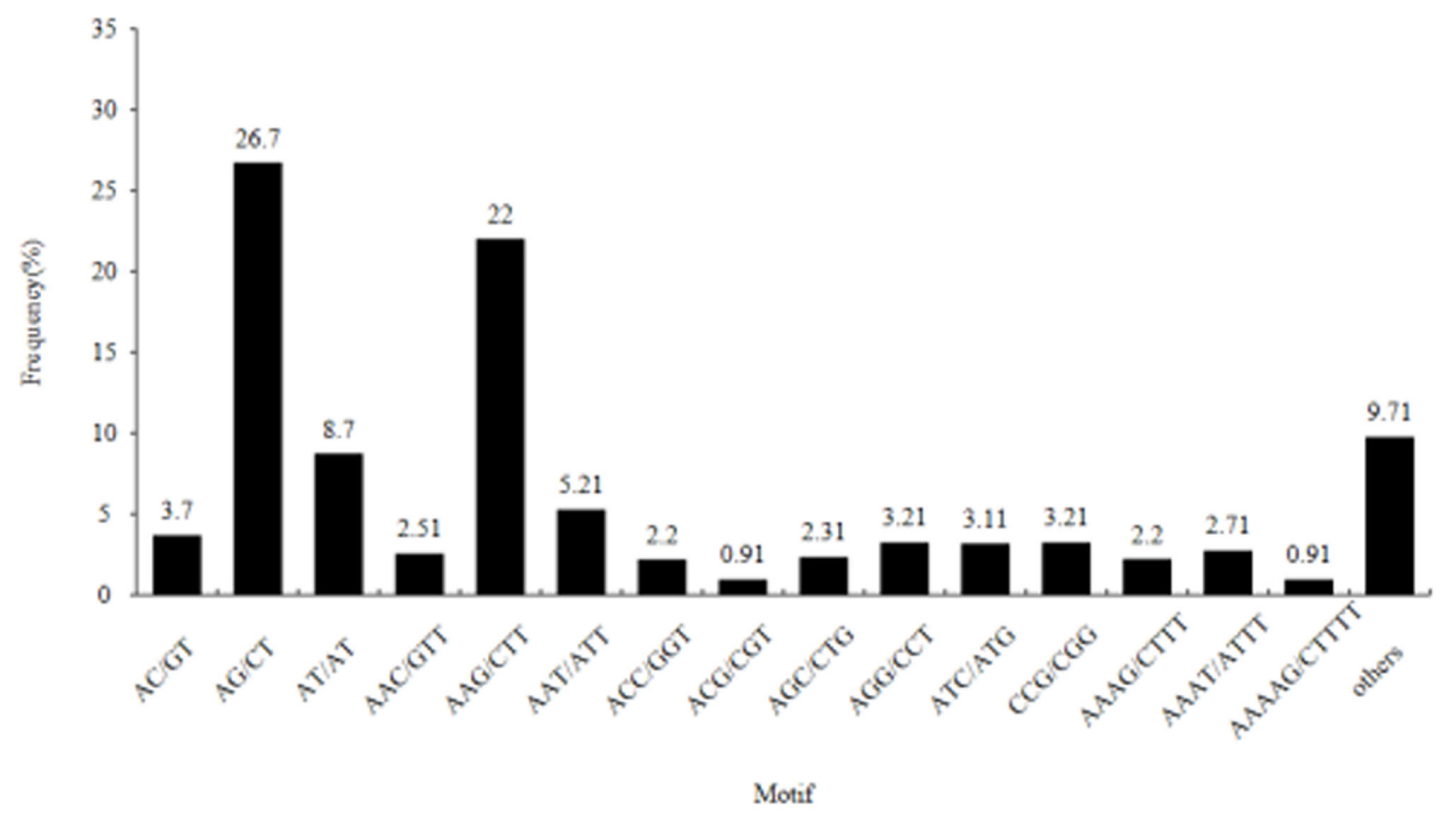
Frequency of classified repeat types of SSRs. The AG/CT di-nucleotide repeat motif was the most abundant one detected in our SSRs.

**Figure 6:**
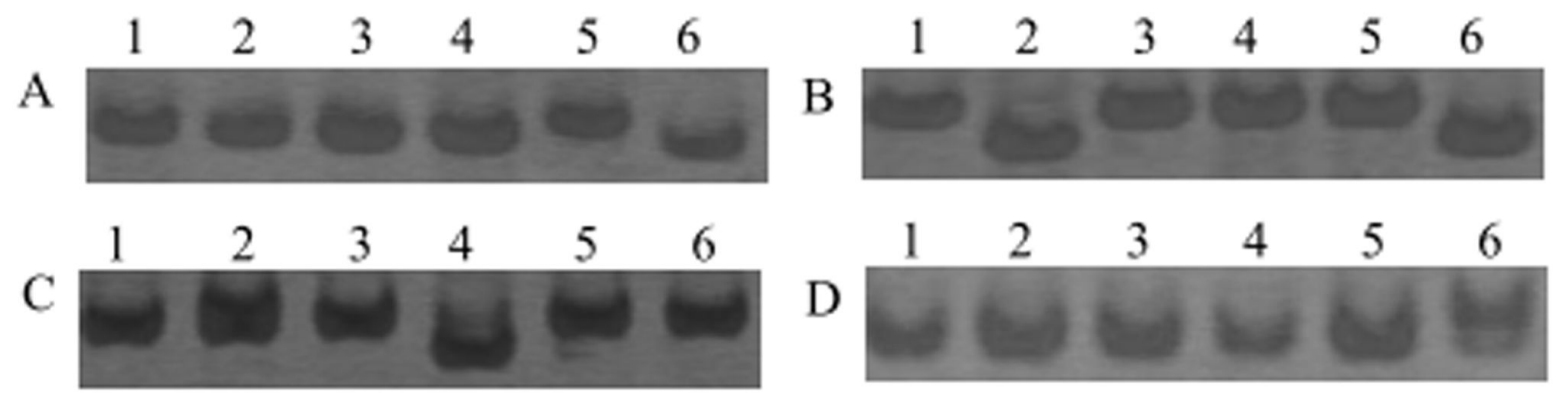
Examples of polymorphic products amplified by SSR primer pairs. A–D. PCR products amplified by primer pair 1, 2, 19 and 35, respectively. 1-6 represent B94, B96, B214, B318, P4, P75, respectively.

Among the searched SSRs, 183 motif sequence types were also identified. The number of di-nucleotide, tri-nucleotide, tetra-nucleotide, penta-nucleotide and hexa-nucleotide repeat types was 4, 10, 24, 49 and 96, respectively. The di-nucleotide repeat AG/CT was the most abundant motif detected in our present study (26.7%), followed by the motif AAG/CTT (22.0%), AT/AT (8.7%), and AAT/ATT (5.2%). And the remaining types of motif accounted for 37.4% (Figure 5). Based on the SSRs, 200 primer pairs were randomly designed to test the amplification effect in six varieties. One hundred seventy of the 200 primer pairs successfully yielded amplification products at the expected size, and 49 (28.8%) of them exhibited polymorphisms among the six varieties. Several representatives of the polymorphic bands were present in Figure 6. 

## Discussion

The lack of genomic information in wax gourd has hindered the research on this important vegetable crop at molecular biology and genetic level. Fortunately, the emergence and availability of the next-generation sequencing technology have provided a powerful and cost-efficient tool to obtain large amounts of transcriptome data from many organisms and tissue types without a reference genome [25,26]. In the present study, we conducted a comprehensive study on the *de novo* assembly and characterization of the transcriptome of wax gourd using this technology and developed a large number of SSR markers based on the transcriptome information obtained. To the best of our knowledge, this is the first exploration of the transcriptome of wax gourd through the analysis of large-scale transcript sequences. These datasets will provide a valuable basis for the future studies on physiology, biochemistry, and molecular genetics on wax gourd.

In order to obtain a maximally informative transcriptome sequence resource of wax gourd, cDNA samples reverse transcribed from total RNA samples that were pooled from shoot tips, leaves, flowers, fruits and stems were normalized prior to sequence analysis. In our present study, more than 44 million high quality reads with 96.1% Q20 bases were generated from wax gourd using Illumina paired-end sequencing technology. The RNA sampled from the above mentioned five different tissues for sequencing could provide more abundant and comprehensive information about transcriptome profiles of this crop. Similar approach was also applied for the study in *Oryzias* *melastigma* [27]. When being *de novo* assembled using Trinity method, 65, 059 unigenes with an N50 of 1, 132 bp were yielded. The average length of the unigenes was 709 bp, which was longer than those assembled in previous studies, for example, with butterfly (197 bp) [28], sweet potato (581 bp) [15], *Salvia* *miltiorrhiza* (331 bp) [19], and safflower (446 bp) [29]. The sequencing depth refers to ratio of the number of nucleotides with the test gene or transcriptome. In this study, the average sequencing depth was about 44 fold. In addition, the coverage depth of the assembled unigenes was also detected against *C. lanatus* orthologs. Most of *C. lanatus* orthologs could be covered by our unigenes (Figure 2). The above results indicated the high quality of our transcriptome sequencing and *de novo* assembly.

For gene annotation, the sequence similarity search was performed against protein databases, including Nr, Swiss-Prot, GO, COG, and KEGG [21,30]. Most of our unigenes could match unique known proteins in public databases, implying that the transcriptome sequencing yielded a great number of unique genes in wax gourd. A large number of unigenes were assigned to a wide range of gene ontology categories and COG classification, which indicated that our transcriptome data represented a broad diversity of transcripts in wax gourd. Similar results were also reported in other species [15,19,27,29,31]. Based on sequence homology searches against the KEGG database, 18, 713 unigenes could be mapped with 281 pathways. Notably, KEGG predictions identified many unigenes associated with the biosynthesis of unsaturated fatty acids, vitamin B6 metabolism, arachidonic acid metabolism, flavonoid biosynthesis, and phenylalanine metabolism, implying that wax gourd is very rich in nutrients, and has important medicinal and health values. Generally speaking, through transcriptome sequencing and gene annotation, such large number of transcriptome sequences will provide an excellent resource for gene isolation and gene expression profile analysis in wax gourd.

SSRs are a group of repetitive DNA sequences that represent a significant portion of higher eukaryote genomes. SSRs are typically co-dominant and highly polymorphic, and are becoming a common source of marker systems for genetic mapping, molecular breeding, gene mapping, comparative genomics, and population genetic analyses in a wide variety of species [32,33,34,35,36,37]. Traditional methods for SSR marker development are expensive, laborious and time-consuming. Luckily, the newly developed high throughput sequencing technique is a powerful and cost-efficient tool for transcriptome sequencing [9]. The transcriptome data was an excellent source for microsatellite mining and SSR marker development, and had been utilized in many species [15,30,38].

In the present study, we identified a total of 6, 242 SSRs based on the transcriptome data of wax gourd. Tri-nucleotide motifs are the most abundant form of SSR repeat structure, which is consistent with that reported in other species [15,38]. In order to assess the quality of the newly developed SSR markers, 200 primer pairs were randomly designed. One hundred seventy of them successfully yielded amplification products at the expected size, and 49 (28.8%) exhibited polymorphisms. Similar amplification rate and polymorphism frequency was also reported in sweet-potato [15] and lentil [38]. Since 6, 242 SSRs were identified in our transcriptome data, more SSR primers can be designed for future research, involving genetic diversity assessment, genetic mapping, and marker-assisted breeding in wax gourd.

In conclusion, the transcriptome sequencing analysis of mix RNA from five different tissues of wax gourd was conducted using Illumina paired-end sequencing technology technique. More than 44 million of high quality reads were generated, and approximately 4 Gbp data were generated, and assembled into 65, 059 unigenes, with an N50 of 1, 132 bp. Most of the unigenes have been sequence annotated. Furthermore, six thousand of SSR primer pairs have been designed using unigenes as templates, and a fraction of them was proved to be effective for polymorphism detection with different varieties of wax gourd. The availability of this ample amount of information about the transcriptome and SSRs in wax gourd could serve as valuable basis for the physiology, biochemistry, molecular genetics and molecular breeding on this agriculturally important vegetable crop.

## Materials and Methods

### Plant material and RNA extraction

One wax gourd inbred line “B98” was grown in the research experiment field of Vegetable Research Institute, Guangdong Academy of Agricultural Sciences, Guangzhou, China. Samples were collected from shoot tips, leaves, flowers, fruits and stems in the flowering stage. The sampled tissues were frozen in liquid nitrogen immediately and stored at -80^o^C until use.

The total RNA of each of above listed samples was isolated using the Trizol Kit (Promega, USA) according to the manufacturer’s instructions. Then the total RNA was treated with RNase-free DNase I (Takara Bio, Japan) for 30 min at 37^o^C to remove residual DNA. RNA quality was verified using a 2100 Bioanalyzer (Agilent Technologies, Santa Clara, CA) and were also checked by RNase free agarose gel electrophoresis and the concentration of the total RNA was measured by a 2100 Bioanalyzer at 260 nm and 280 nm. Only those RNA samples whose 260 nm/280 nm ratio was between 1.8 and 2.0 were used for subsequent analyses. Equal amounts of RNA from each sampled tissue were mixed for the subsequent steps of our experiments.

### cDNA library construction and sequencing

In brief, poly (A) RNA was collected from 20 µg of total RNA using Sera-mag Magnetic Oligo (dT) Beads (Illumina). To avoid priming bias when synthesizing cDNA, the purified mRNA was first fragmented into 200-700 bp by fragmentation buffer. Then the cleaved RNA fragments were transcribed into first-strand cDNA using reverse transcriptase and random hexamer-primers (Illumina), followed by second-strand cDNA synthesis using DNA polymerase I and RNase H. The double-stranded cDNA was further subjected to end-pairing using T4 DNA polymerase, the Klenow fragment, and T4 polynucleotide kinase followed by a single <A> base addition using Klenow 3’ to 5’ exopolymerase, then ligated with an adapter or index adapter using T4 DNA ligase. The products of adaptor-ligated fragments were separated on an agarose gel and a size range of templates were selected for downstream enrichment. A range of cDNA fragments (200 ± 25 bp) was excised from the gel and purified. Using these purified cDNA as templates, a paired-end library was constructed using the Genomic Sample Prep kit (Illumina), according to the manufacturer’s instructions. The cDNA library was constructed with a fragment length range of 200 bp (± 25 bp). Finally, the cDNA library was sequenced on a PE flow cell using Illumina HiSeq^TM^ 2000. After the first read was completed, the templates were regenerated *in situ* to enable a second 75 bp read from the opposite end of the fragments. Once the original templates were cleaved and removed, the reverse strands underwent sequencing-by-synthesis.

### *De novo* assembly and gene annotation of Illumina reads

*Do novo* assembly was carried out using Trinity method [26]. Finally, 44, 925, 792 sequencing reads with 75-mer length were obtained. The sequencing data have been deposited in NCBI Sequence Read Archive (SRA, http://www.ncbi.nlm.nih.gov/sra) with an accession number of SRA074508. The reads were first combined to form longer fragments, i.e., cotigs. The reads were then mapped back to the contigs, and the paired-end reads and contigs from the same transcript were assembled to form a longer sequence, with N for unknown sequences, i.e., scaffolds. Paired-end reads were again used for gap filling of the scaffolds to obtain Unigenes with the least Ns that could not be extended on either end. To evaluate the depth of coverage, all reads were realigned to the unigenes using SOAPaligner (http://soap.genomics.org.cn/soapaligner.html) [20]. BLASTN was used for comparison of transcriptome and the CDS sequences of *C. lanatus* (E value <10^-5^). According to the colinearity principle, the match results of BLASTN were combined. The best comparison result between each transcriptome unigene and the CDS sequence of *C. lanatus* was determined, and these two genes (one is from the transcriptome assembly, another from the homologous species) are homologous to each other.

All of the unigenes were then compared with protein databases, such as NCBI non-redundant protein (Nr) database, Swiss-Prot protein database, the Kyoto Encyclopedia of Genes and Genomes (KEGG) pathway database, and the Cluster of Orthologous Groups database, with an E-value cut-off of 1e^-5^. The best aligning results were chosen to decide the direction of unigenes. Based on the results of protein database annotation, Blast2GO [30] was employed to obtain GO annotation according to molecular function, biological process and cellular component ontologies. The unigenes were also aligned to the COG database to predict and classify possible functions. In the meantime, the KEGG database was used to annotate the pathway of these unigenes with E value threshold of 10^-5^ [21].

### SSR mining and primer design

The MIcroSAtellite (MISA, http://pgrc.ipk-gatersleben.de/misa/) was employed for microsatellite mining. In this study, the SSRs were considered to contain motifs with two to six nucleotides in size and a minimum of 4 contiguous repeat units. Based on the MISA results, Primer premier 6.0 (PREMIER Biosoft International, Palo Alto, CA) was used to design primer pairs in the flanking regions of SSRs, and the PCR product size was ranged from 100 to 280 bp. A total of 200 primer pairs (File S1) were synthesized and six varieties were selected to validate the polymorphism of the SSR markers tested.

## Supporting Information

File S1 Primer sequences of SSR markers.
(DOC)
