# Supplementary material for: De Novo Assembly and Characterization of the Transcriptome, and Development of SSR Markers in Wax Gourd (Benicasa hispida)
Source: PLoS One. 2013 Aug 8;8(8):e71054. doi: 10.1371/journal.pone.0071054 (PMC3738631; doi:10.1371/journal.pone.0071054)
Supplement: File S1 — Primer sequences of SSR markers. (DOC) [file pone.0071054.s001.doc]

**Table S1.** Primer sequences of SSR markers

| Primer code | Repeat motif | Forward primer (5’---3’) | Reverse primer (5’---3’) | Product size |
| --- | --- | --- | --- | --- |
| 1 | (TC)6--(CTTCGT)4 | TTCGTTGTTGCTGTTTCTGG | TCACGGAACAAAATGGGAAT | 168bp |
| 2 | (TTC)5 | ACCACTCCCCTTTGTTCCTT | GGATGTGAGGAATGCGACTT | 268bp |
| 3 | (AGA)6 | CGACGAACAACATGAACACC | CTACCCATTTCCCCATTTCC | 199bp |
| 4 | (TC)7 | CCTCCTCGAATTTCTGTTGC | AAAGCCTTTGCTCTGTACGC | 241bp |
| 5 | (TA)10 | CCCATGTCTTGTTTGGGACT | AATTCCGGGTAGGTTTTTGG | 142bp |
| 6 | (ATA)7 | GCCACCACACACAATCACTC | AAACCCCTCCAAATTGCTCT | 272bp |
| 7 | (AAT)7 | AGAGCAATTTGGAGGGGTTT | CCTTGCCAAGTGGATTGATT | 216bp |
| 8 | (CGG)5 | GTATCGGCATCATACCCACC | TGAGATGGTTGGATTTGGGT | 211bp |
| 9 | (AAG)6 | CAACAACACAATGAGGAAGAAGA | GGATTCCGATGACCCCTATT | 237bp |
| 10 | (TTAT)4 | TGGGGATGATGTTGAATTTG | ACACCTCAAGGCGATGAGTC | 236bp |
| 11 | (TCCACC)4 | AAACGAAATCAAGCAAACCG | GTGGTGGTGGAGTCCCATAC | 227bp |
| 12 | (CCA)6 | ATGAGGCTCCTTCATTGCC | GCATTGGAGGAGGTGGTG | 261bp |
| 13 | (TAA)5 | AAGCAGATGGCTGCTGAGAT | GTGAGATCGTGGGAGAGAGC | 211bp |
| 14 | (CCG)6 | CTATTGGAGAAGCCGCTGTT | TAAGAGTTGAGCGCTGACGA | 164bp |
| 15 | (AAGA)7 | GAACGGGCAAATTGAAAGAA | CACATCCCCTTCAGAGTTGG | 210bp |
| 16 | (CAA)5 | TTGGCCCCTCTCTAATTCCT | GGACTGACACGTGGAGGTTT | 143bp |
| 17 | (CTT)5 | TCCTATCCCGTGATCGATTT | GTCTGGTCGGCTTGATTGTT | 205bp |
| 18 | (TAA)5 | AAGCAGATGGCTGCTGAGAT | GTGAGATCGTGGGAGAGAGC | 193bp |
| 19 | (TC)6--(CTTCGT)4 | TTCGTTGTTGCTGTTTCTGG | TCACGGAACAAAATGGGAAT | 168bp |
| 20 | (TAA)5--(GCCGGA)4 | TCGAGGCACAAGGGAAGTAT | AAAAGAAGGGAAGCCGAGAG | 218bp |
| 21 | (CACCGA)4 | TCTTGATTCTGTGCAGTGGC | GACTTGCACAGTGGCTTTGA | 271bp |
| 22 | (TGC)5 | GCCCAATTCTTCAAACCAGA | TCCTTGAGAAGCCGTGAACT | 125bp |
| 23 | (TCATAT)4 | ATTTCGTCAACGCCATTAGG | TCGCTCCGGTGATTTTAGTC | 207bp |
| 24 | (AAT)5 | AATTTTGGTCCCTGAACTTCTAT | CCAGAGTTGGCTTGAGCTTC | 229bp |
| 25 | (TGTTCC)4 | TCCAGACATCACTCCACCAA | GAACCGGAACGGTAACTGAA | 209bp |
| 26 | (GCCTGC)4 | AATGTTTTAGGCCCCCAGTC | CCCCTCCTCTTGTACTGCTG | 169bp |
| 27 | (GAA)5 | AGCAAAGTAATGGTGGCTGG | TCAGAACTGTCCCTTCTCTTCC | 242bp |
| 28 | (TGA)5 | TCGCCCAGGAAATATTCAAG | CCCCTCACTATCTCTTCGACA | 211bp |
| 29 | (TGT)5 | GCTTCTTCATCATCAATTTCCA | TCAGTTGGCTTAGCCTTGGT | 169bp |
| 30 | (TCC)5 | AGCATGCGAACGTAACTGC | GGGAATGATGCCTGAAGTACA | 226bp |
| 31 | (ATCT)4 | CTCAAAATGAAGGTTATTGCCA | TGTCTTTGATCTATGTGCCTGTG | 154bp |
| 32 | (CT)6--(GAA)6 | GGAATTTGGATTGGAGAGGA | GTCTGGTTGAAGCTCCCAAA | 261bp |
| 33 | (ACC)5--(TAA)5--(ATA)6 | AAGCCTGTTGCTCCCAGTAA | CGTTTTCGTCGGTTGTTCTT | 255bp |
| 34 | (AGAGA)4 | AATGTGAGAAGAGCCCCCAT | CGAGGACTACAATGGCGTTT | 121bp |
| 35 | (TTTCAA)5 | TGGGAGAGAAATTATGGGGA | ATTGAGGCCACAAACCAAAG | 157bp |
| 36 | (TC)7--(AG)7--(GAA)5 | AATGCAAGGGCAAAGAGAGA | CGTTTTGACCAAAGCTTTCAC | 222bp |
| 37 | (GTGGG)5 | ATGTGTGTGCAGACTGGCTC | AGCTTGCGGATTTTCTTCAA | 188bp |
| 38 | (GAA)5--(AAG)6 | TCGCTTCGACTGAGGAAGAT | TTTGTTCAAACATCCCACCA | 255bp |
| 39 | (CTT)5--(CCT)6 | GTTTCTCTCCGTTCCACCAA | ATCGCTTCTCGTCAACTCGT | 243bp |
| 40 | (TC)9-- (TC)6 | TGCGAATTTCAGGGTTCTCT | TTTGGGTCAGCCTCCTACAT | 173bp |
| 41 | (GAA)5(AG)6 | TCGAATTGGATCTTGGGAAG | GCCTCATTCGTGCTGGTAAT | 167bp |
| 42 | (AT)9 | TGCTGACTTTGACGTTCCAG | TCAGAGGTTCAATCTCCCGT | 274bp |
| 43 | (TAT)6 | AATTGGCAATGTTCTCCTGG | CGGCAAATCAGTGGAAAGAT | 275bp |
| 44 | (AAAT)4 | GGGTTAATTATTGTTCATTACCCAA | TGTGTTTGGGGTGCAAGTTA | 269bp |
| 45 | (TCG)5 | GGGATATAAGCAGGGCTTCC | ATAAAGACGGATGGCTGCAC | 144bp |
| 46 | (AGA)5 | GGCTTTTTCACAGCAAGGAG | ACCCTCCCAATTGTTTCCTC | 149bp |
| 47 | (AAC)6 | CAGCAAAAAGCAGTCATTTCC | TCGGCCTTTTTCTTTCTTCA | 152bp |
| 48 | (TTTG)6 | TTTTTCCCCCATGTTGTGTT | AGAAACAAAACCCCGAAACC | 259bp |
| 49 | (TCT)6 | TTGCTCAACTTCAGTGGCTG | GCAGCTGTTCGGTTTCTGTT | 247bp |
| 50 | (TC)6(AC)12 | CCAGCTCGGTTCAACAAAAT | TCCATTTTTGTTTTCTGCCC | 135bp |
| 51 | (GGC)6 | CGATCTTCAGGACCTTGCTC | GGAGGGTTTAATTCGACGGT | 258bp |
| 52 | (TTTTC)4 | ATCAATATCTGCACCAGGGC | AACTACAATGGAGGGGAGGC | 270bp |
| 53 | (GACAGT)4 | AAAGCCAAGGATGATGTTCG | TTATCCCTGCCACGAGATTC | 275bp |
| 54 | (TTGCAG)4 | TCGCCGATAAACCCATAAAC | GGCACCAGAAAGAGAAGTGC | 258bp |
| 55 | (CCG)5--(CGACCA)4 | TCCTTCCCTTCTTCCTCCAT | AGATCTGGGGACATCCTCCT | 273bp |
| 56 | (GTTT)4 | TATTACGTGTGTGTGCACGG | AGCCGGATGACAAAATTCAG | 225bp |
| 57 | (TCCT)4 | TAACTCCCAACTCTGTCGCC | TACGCCTTTTTACTGCCACC | 182bp |
| 58 | (AATC)4 | CTTCTTCGTCGTCTTCGTCC | ATTGGGGAATACCCATTTCG | 167bp |
| 59 | (TA)8--(AAAT)4 | CACGTCATCATCATACATGTTTCT | CACTCTCCCTCTCTTGTGGG | 200bp |
| 60 | (TGA)6 | CCATTTCCATGGATTCTGCT | ATCGTATCCATGGTGGCCTA | 278bp |
| 61 | (TAT)6 | AACTCCTCCCAAATTCCCAC | GAATGTTGTTTTGGTTGGGG | 205bp |
| 62 | (CCAAT)4 | TTTCATGGTTGTTTTGGCTG | GGCCGCAGTATATAGGCAGA | 186bp |
| 63 | (TTTA)4 | GGGCATTTCATTTTCAGCTT | GCAAAAGAAGAAAATATTTCAAGCA | 105bp |
| 64 | (CAG)6 | CCCAGTTCAATACCCCCTTT | CCGAGAGTTTCCGTACAAGC | 194bp |
| 65 | (GAA)5gtg(GAA)5 | TCCCCTTCTCAGTTCCCTTT | CCCATCTTCCTTTTGCTGAA | 204bp |
| 66 | (TAT)5 | TGAACATCATGCTAGTTGAGTTACAC | CCCACTCCACCTACCAGAAA | 149bp |
| 67 | (AAAT)4 | CATTTCCACAATATGCACAACA | TGGAAGTTTTTCCAAATGGC | 257bp |
| 68 | (AAAAT)4 | CCAAAATAAAATTGGTTAACATCCA | TTGAGATGATTAGGTGTGCCC | 100bp |
| 69 | (TAT)6 | CATTTCAATCAGTTTCAACTTCCA | CAGCTCCTGCAGTGAAAGAA | 124bp |
| 70 | (ATTTT)4 | TCACTGTCATTGATTCAGGCA | CCACTTTCGCTAAATGATTCC | 279bp |
| 71 | (AGC)6 | ATCAGCAAATGCAAGCAGTG | AAACGGGTTGATGATTGAGG | 234bp |
| 72 | (CATA)4 | GGGGGTGTTCTTTTGTTTGT | TCTTTATGAAAGTTTGGTTTTTCA | 129bp |
| 73 | (TTG)6 | GGTGGCCGGAGGAATAATAA | ATTGGCCAGCAATTTCATGT | 251bp |
| 74 | (TTTC)4 | CAATTGGTTTTCCAACCACC | TCGAATTTGGGTTTAGGGTTT | 160bp |
| 75 | (AAAC)5 | GCCACCGAATTTCAAGCTAA | ATGAGTCATCCCTCCCCACT | 251bp |
| 76 | (TTTA)5 | ATGCTCCTCCTCCCTCATTT | TTTGCTTGGGAGATTGAACC | 117bp |
| 77 | (GCT)6 | GCTGCAACCTTATGATCCGT | TCAGGGCGGAAGTTTAGAGA | 253bp |
| 78 | (ATTA)6 | AAAAACCAAAACCCCGAAAG | ATGGTTAAACGCTAAGGGCA | 184bp |
| 79 | (CTCCGA)4 | ACCTCTCAGCTCCTTCACCA | TATGCTGATTCTCGTTTGCG | 115bp |
| 80 | (CTC)5--(TC)6 | CGTTCCAGTTCTTCTCCAGC | CGAAGTGGAAAAGCGAAAAG | 235bp |
| 81 | (AATG)4 | GACAGAATTGGGGGTTTGTG | TCAACAGCAGTTGGTGGAAG | 188bp |
| 82 | (AGA)6--(TC)6--(TC)6 | TCTTGATGGGTGTTGGTGAA | ATTCTTGACGGATTTGGCAC | 245bp |
| 83 | (AT)10 | TTTGATTTGCTTTAGTTTGAAAGAA | GTGATAGGAGGTTCAGCCCA | 143bp |
| 84 | (TAAA)4 | ATGCTCCGTATGTGCTCCTT | ACCCTGCACCTCATAACTGG | 147bp |
| 85 | (AAAG)4 | CAGAACACGGACTCACTGGA | CTAACCCTGTGGACTTCCGA | 245bp |
| 86 | (TTACGA)4 | TGTGGAGTGAATTCATGCGT | CTGAAAGCAAACAATCGCAA | 119bp |
| 87 | (TATC)5 | GAATCAGAAAACAGCTTTATATGCC | CGGGCTTCAATTTCAAAGAC | 272bp |
| 88 | (TCG)5tcctcc(TCA)5 | TGGGTGATGACTCTCCATTG | TGGAGGGAAGATATTGTGGG | 230bp |
| 89 | (AGAAC)4 | CCCACAATATCTTCCCTCCA | GCGCCTGTGAAATCTGTTCT | 235bp |
| 90 | (TTCT)5 | AAACGGAGTTCAACGGTGTC | ACTGCCTTCCACAACCTCAA | 235bp |
| 91 | (CGAAT)5 | CTTTTCCTCTCCCATAGCGA | CAGAGAAAGAAATCCGCAGG | 258bp |
| 92 | (TCT)7 | TCACAGTGGAAGACACAGGC | AGAGGAATCGAGGATCAGCA | 242bp |
| 93 | (GAT)6 | ATGAAGGGGTTTTGCAGTTG | GGGAAGATTTCGCTTCCTCT | 251bp |
| 94 | (ATA)7 | GCCACCACACACAATCACTC | AAACCCCTCCAAATTGCTCT | 272bp |
| 95 | (AAT)7 | AGAGCAATTTGGAGGGGTTT | CCTTGCCAAGTGGATTGATT | 216bp |
| 96 | (TTA)6--(TA)6 | GATCCATCCACTCTCTTCCAA | AGTTGGTCGGAATCTGATCG | 219bp |
| 97 | (ATA)7 | GCCACCACACACAATCACTC | AAACCCCTCCAAATTGCTCT | 272bp |
| 98 | (AAT)7 | AGAGCAATTTGGAGGGGTTT | CCTTGCCAAGTGGATTGATT | 216bp |
| 99 | (AAG)6 | GAGCCCAAAACTGAAATGGA | TAATGATGATCGGCGTCGTA | 155bp |
| 100 | (GCG)6 | TGAGGAGGAACATCGTAGGG | ATCTTGCTTAAGGCATCGGA | 205bp |
| 101 | (TGGGGA)4 | CAAAACCTTCTTGAGGAGCG | CTCGCGCTCTTTCTCAAAAC | 140bp |
| 102 | (CAAAT)4 | TTCGCCATTGAAATTCCTTC | GTCGGCAGAGGAAGAAGATG | 164bp |
| 103 | (TTA)6 | TATCCCTGACCAACCTGAGC | AACTGACCACTTGCATTGACC | 204bp |
| 104 | (AGTG)5 | ATGGGTGTTTAATGCTTGGC | TCCAGGTCCACAAACTCACA | 138bp |
| 105 | (AGC)6 | AAAAGACAAGCAGCGGAATG | ATCAAACTGCTTCACCCTCG | 175bp |
| 106 | (CAA)6 | GGGTTGATCCCTAATGGCTT | CGGGGACATTTCTTAGGGTT | 218bp |
| 107 | (CTCG)5-- (TC)8 | CGCAGGTAACGCAGAGAAAT | CGCGATAGGGAGAGAGAAAG | 232bp |
| 108 | (AGG)5 | GAGCTTCGGAAAGTGACGAC | CCGCCATCAGTTTCGTTAAT | 236bp |
| 109 | (AAT)5--(AAT)5 | GCTCTTGGCTTTGGCTCTTA | AGCCACAGCCCTCATGTACT | 280bp |
| 110 | (CCA)6 | ACCCCATATTTCTTGTCCCC | AAATGGGTGTTTAACGTCGG | 134bp |
| 111 | (AAG)5-- (CAA)5 | GTTGCGGATGTGGAGAATTT | CCAATCCACCAAAAATCCAT | 150bp |
| 112 | (AT)10 | CTCAATGCCGAGAAGAGACC | TCACACAAAAACCCACTTCG | 259bp |
| 113 | (AAC)6 | TTCACCCTTCTTCTTCCCCT | CCTCAGTCCATCTAGGCAGC | 249bp |
| 114 | (ATGAAG)4 | AAGAGTGGGAAGTGCTTTGC | ATTGGCTCCCATGTGTCTTC | 274bp |
| 115 | (AATA)4 | TGGTGTTTGAAGCCATTCTCT | CCAACAACAGCAACAAAGACA | 239bp |
| 116 | (TTAT)4 | TTAGGGTTTCGTGGAACGAC | TGGAAATTGAGGGTCTCAGG | 243bp |
| 117 | (GCC)7 | TCCCTCTCTGTTGCCAACTT | TCTACAGACACCGAGGGAGG | 242bp |
| 118 | (TTTA)4 | CGGAGCAACAAGCCAATTAT | CGCAATCTTTTCATCCAACA | 253bp |
| 119 | (TTTA)4 | AGCCGGCATAACTACACATAA | CGCAATCTTTTCATCCAACA | 166bp |
| 120 | (GA)8--(AAAG)5 | GCCTTAGGTCCACGAGTTCA | CTGGTGGTCGCTGGTAAAGT | 182bp |
| 121 | (CAAC)4 | TGAATGGCAGGCAAGATACA | TGCTCTTCAGCGCTTTGTAA | 234bp |
| 122 | (GCGTG)4 | GGTACGTGGGTTCGAACAAT | ATTTCTTCTCCACGCTGCAT | 144bp |
| 123 | (AG)8 | AAACTAAAAACCTCCGCCGT | CAGCTGTCGACGGTTACTGA | 273bp |
| 124 | (TTC)7 | AACTCCGGCGACTTCTTCTT | AAATGCCAAAACAACCAAGC | 274bp |
| 125 | (TAT)6 | TGTGGGGACTTGGAGTTTTT | TGATACTTCCTGCTCACACCA | 190bp |
| 126 | (CT)8 | CAATGGAGGTCCTTCCAAGA | TTAGCAGAGGAAGCAAAGGC | 214bp |
| 127 | (TTCCTC)4 | ATGGACCTGTAGGAACGTCG | GAGAATGTTCCGAGGGTTGA | 157bp |
| 128 | (GCCGTT)4 | TCTTTCTTCTCTGCCTCCCC | GGCCATCCAGTTATTAGGGA | 182bp |
| 129 | (AAAT)5 | GGGCCAAAATGATGAAGGTA | ATTCTCGCAAAACCCACAAC | 115bp |
| 130 | (AAG)6 | CGGAGACCAGACAGGAGAAG | AAACCCCATTTCCAATCCTC | 183bp |
| 131 | (CACCGA)4 | TCTTGATTCTGTGCAGTGGC | GACTTGCACAGTGGCTTTGA | 271bp |
| 132 | (TTC)6 | ACAAACCCCATCTCTTCCCT | GCATTTGGGGTTTTGAGAGA | 217bp |
| 133 | (GCC)7 | GGAGGTTGGGTGTAAAAGCA | AGTTTGCTGTTGGTTTTGGG | 143bp |
| 134 | (AGT)6 | GAACGCAGTCTCCTCGATTC | TTGACGGTGTGAAACTCTGC | 199bp |
| 135 | (TTTGC)4 | AGGTACAATGCTCCATTGCC | CAGTTGCACCATGATAAATGA | 149bp |
| 136 | (GGCATG)4 | TGATGGGTTCAGGTATGGGT | GAGGTTGCTGCATTTGGATT | 225bp |
| 137 | (GGCATG)4 | CTAAGGCCGACTTCAAATGC | TGCCCCATACCCATATTCAT | 167bp |
| 138 | (TG)11 | ATTCATATGCCAGAAAGGGG | GGAAACCCCATCAACCTTTT | 266bp |
| 139 | (GCC)7 | GGAAGGGAACCTAAAACCCA | GCTCCTCTTCACCATCTTCG | 235bp |
| 140 | (GCC)6 | GAGCTTCCATTTTGGCTTTG | TCCGAGTTTGGAGATTTTGG | 237bp |
| 141 | (CTTCAG)4 | GAATGGAGAAGCGGTTGGTA | GCAATGGAGGATATGATTGGTT | 212bp |
| 142 | (CTGCAC)4 | CTCCTCTGAGAGCTTGGTCG | ATGGCACAGAGGAAGGAAAA | 250bp |
| 143 | (TA)8 | GAAAACGAGAAAGCACTGCC | TTGGCGATTTTGGTAGGTTC | 160bp |
| 144 | (AGA)5-- (GGA)6 | GCTTGTGGGTTGGGAAAATA | CAGTGACAGAGCATCCATCG | 242bp |
| 145 | (TTC)6 | AACCCTCAACAACAACAATGC | TTTTCCAGGTTTCCATGAGG | 167bp |
| 146 | (TTCTT)4 | TCTCTCCCATATTTTCCCCC | TGGGAACAAAATCCAGAAGC | 147bp |
| 147 | (TCT)7 | TCAAACCAGCAATGGATGAA | GGCTGTTGGTGGTCGTAGTAA | 143bp |
| 148 | (CCG)6 | CTTCCAAGGACGCTTACCCT | ATCCTTCGAGGCAACCAGTA | 156bp |
| 149 | (AAAT)4 | TAAACTCGGCAGCACATTCA | GGAATCAATGTTGGATTGGG | 255bp |
| 150 | (TGG)6 | GATGATGGGGTTGGTGAGAC | ACCGCCATGGCTACATAATC | 250bp |
| 151 | (AAAAG)4 | GAGGGCAAGAACTAGACATAAGTG | GCCCCTAGAAAATAGGCTCG | 182bp |
| 152 | (CT)10 | GCTGGAAAAGAGAAAACCCC | ACGCGAGCGAAGTCTATCAT | 161bp |
| 153 | (TAAAA)8 | TGTCAGGCTGTCAAAACCAA | CAATCCGCCATTGTTTTCTT | 272bp |
| 154 | (TTTCCT)4 | TCCATATGCCATTAGCCCTC | AAAGTGGAATCAAAAGGGGG | 206bp |
| 155 | (TTTC)4 | TTCGCTTGGATTTCTGAACC | GGTCTTTGTTTCTGTTCTGGG | 181bp |
| 156 | (CAGT)5 | CCAGAACAAGAAAACAACTCCA | TTTGCTTCTTGAACTTGGGG | 170bp |
| 157 | (TTC)6 | CCGTCAAGCTCTTTGTCCTC | GCTCAAATCCTCCATTTCCA | 184bp |
| 158 | (TTC)6 | TTCCGATTCCATTTCCATTC | CCACGCTCAGCATACTTCAA | 276bp |
| 159 | (GCT)6 | ACCACCAAGACTTCCCTGTG | CTTCAGTTTATGGGCCAGGA | 242bp |
| 160 | (AT)8 | GGAGGTGGAGATGATTTGGA | AAGGTGGGAAACTTTAGGCA | 274bp |
| 161 | (GAGAA)4--(GAT)6 | TGGACCTTGGAAAGTGAAGG | CCCAAAATTCTCCCCAAAAT | 197bp |
| 162 | (TTTTC)4 | CTTGAGCAGCCATGAATCAG | TTGTGCAGAACACACACCAA | 226bp |
| 163 | (GAA)6 | CGGATTCAAATTAAAGCGGA | GGAAATCAGATTGGGGGAAT | 134bp |
| 164 | (CTT)6 | TCCATGGCTTCTTCAAATCC | AGCGAGTTCGATGCTGTTTT | 219bp |
| 165 | (GAA)6 | TTGAAAGGAGGTTGTTTGGG | TCTTCCTCCTTGCTACCCCT | 223bp |
| 166 | (TCAC)4 | TGAGGGCATGATGAACAAAA | GGCATAGGGATTGGGAATCT | 237bp |
| 167 | (TTTCAC)4 | GACTCTTTACCCATTCCCCC | ACGATTGAGTTCCCCAGTTG | 252bp |
| 168 | (AT)8 | GCAGCAGAATAGCCATGTCA | TGATACATCAGGGGGCTTACA | 247bp |
| 169 | (TC)8 | CGGTGACACTATCCGGTTCT | GGCAAGCAGAAAACTTGGAG | 207bp |
| 170 | (TCA)7 | TGTCTCTTTTGCAATGTCCG | GCCCCATAAGAGATTGGTGA | 187bp |
| 171 | (TCTA)5 | AGGAGCAGCAGCAGTATGGT | GCACACTTCAAATCCCATCC | 278bp |
| 172 | (AAT)7 | ACAGCATGCGTATCGATGAA | GCAGGCAAAATCTTGAGGAA | 186bp |
| 173 | (TACC)4 | GGTTCCCATGTGTCTTCCAC | GAAAAAGCGGCTTCTCAATG | 193bp |
| 174 | (TTA)6 | GAGAATTGTGCCGCTGAGAT | CGGTTCATTTAGCACACCAA | 253bp |
| 175 | (AG)10 | AAAATGGAACACTTTGGGGA | GTTTGTCGTGGTTGTTGTGG | 199bp |
| 176 | (AAG)5-- (GAA)6 | GACCAACATCGTGTAGCCCT | TTGCAAGTTCTTCCCAACAA | 243bp |
| 177 | (GAA)6 | ATCCATCGGATTTTGGATCA | TGGTTGGGACAGCATATGAA | 274bp |
| 178 | (TGGC)4 | GGAGCCTGTAACGTACCGAG | CCGCTTCTATAATGGACCGA | 161bp |
| 179 | (CCG)6 | CCATTCAGCCGTGTAAACAA | TGGTGGGAAAGAAGGTTGAC | 198bp |
| 180 | (GAA)6 | AGGGAGTGAAGCCCAAGAAT | CGGTCAAAACAGAGTGCAAA | 185bp |
| 181 | (GAA)6 | ATAGGCCGATTCGGAGAAAT | ATGCCAAGAATCCAAACACC | 236bp |
| 182 | (TTTTC)4--(ATC)5 | CTCAGATCCGAGTTTGGAGG | GGAATTGGGGAAGCAAAAAT | 266bp |
| 183 | (TCT)6 | AGACCTTATGACGACGGTGG | AAAGGCAGAATATGGCATGG | 274bp |
| 184 | (CGACC)4 | TTCCCGCAAAATATCGAAAG | TGGGCATTACCACAAACAGA | 160bp |
| 185 | (TATT)4 | TCGTGAGGTCTTTGGTTCAA | TCCGAAACTATGTGGGCTTC | 186bp |
| 186 | (AGA)6--(AGA)5 | GGCCATCTGAGAGATACCCA | CCCCTCATCTAATCCCCAAT | 141bp |
| 187 | (AGA)6 | ATTGGGGATTAGATGAGGGG | GCCACGGAGAGAAATGAATG | 253bp |
| 188 | (TCTT)4 | GTCCACGGTGAGGTAGGAAT | TTTAGAAACCCCAACATGGC | 275bp |
| 189 | (GCC)6 | ATCGATGAATGGATTTTCGC | CGATCCAAGGAATCGTCTGT | 194bp |
| 190 | (CCG)6 | ATACTTTTTCCGGCTACCGC | ATAAGGAGCCCTGTGGTGTG | 197bp |
| 191 | (GCG)7 | GCTCGTTCCTGGTCGAGATA | CAGCTACCGTTTCTCCCTTG | 227bp |
| 192 | (TA)7--(TA)8 | CAAGCTCCAGGGAGAAGTTG | GTGGATTATATGGGGGCCTT | 245bp |
| 193 | (GCTG)4 | TGCATTCAACTTTGCTTTCG | CCTCCAATCCATGCTCACTT | 264bp |
| 194 | (TTCC)4 | CAAAGAAATCCCAAGAGGCA | GTGGGTGGGAAGAACAAGAA | 191bp |
| 195 | (TC)9 | ATTGCATCAGAAATCGGGAG | TTTGAAGTGGAATTCGGAGG | 157bp |
| 196 | (TAC)6 | CCTCTTCCTCCTCCATTTCC | CAGTTGTGTGGCCTCTTGAA | 259bp |
| 197 | (CGC)6 | TAATGGCTTCCCAAGAATCG | TTCCATCTGGGGTTCTCTTG | 280bp |
| 198 | (CT)9 | TGCAGCAACTGAAATGAACA | GCAGAGGCTATGGAAAATGC | 154bp |
| 199 | (CT)8 | CCCCATCGTTTACCTCTTCC | TGTTGTCGGTCTCCACCATA | 179bp |
| 200 | (GCA)6 | CAGACAGCACAACAGCGATT | CAAGAGCCAACGAGTCCTTC | 265bp |
